# Sex does not clinically influence the functional outcome of total knee arthroplasty but females have a lower rate of satisfaction with pain relief

**DOI:** 10.1186/s43019-020-00048-1

**Published:** 2020-06-17

**Authors:** N. D. Clement, D. Weir, J. Holland, D. J. Deehan

**Affiliations:** Department of Orthopaedics, Freeman Hospital, Newcastle upon Tyne Hospitals NHS Foundation Trust, Freeman Road, High Heaton, Newcastle upon Tyne, NE7 7DN UK

**Keywords:** Sex, Total knee arthroplasty, Outcome, Satisfaction, WOMAC

## Abstract

**Background:**

The aims were to assess whether sex had a clinically significant independent influence on the outcome of total knee arthroplasty (TKA) according to the Western Ontario and McMaster Universities Osteoarthritis Index (WOMAC) score, Short Form (SF-) 12 scores and patient satisfaction at 1 year.

**Methods:**

A retrospective cohort of 3510 primary TKA were identified. Patient demographics, comorbidities, WOMAC and SF-12 scores were collected preoperatively and 1 year postoperatively. Patient satisfaction were assessed at 1 year.

**Results:**

There were 1584 males and 1926 females. The preoperative WOMAC and SF-12 scores were significantly (*p* < 0.001) worse in females but were not greater than the minimal clinically important difference (MCID). When adjustments had been made for confounding differences, females showed a significantly greater improvement in their function (1.5 points, *p* = 0.03) and total (1.5 points, p = 0.03) WOMAC scores compared to males, but these were not greater than the MCID. When adjustments had been made for confounding differences, females were less likely to be satisfied with their pain relief (*p* = 0.03) relative to males.

**Conclusion:**

Sex does not clinically influence the knee specific outcome (WOMAC) or overall generic (SF-12) health 1 year after TKA. However, satisfaction with pain relief after TKA was significantly less likely in female patients.

**Level of evidence II:**

Prognostic retrospective cohort study.

## Introduction

A higher prevalence of osteoarthritis exists in females relative to males, which is reflected in a greater proportion of females undergoing total knee arthroplasty (TKA) per year [1]. Numerous factors have been shown to influence the functional outcome and satisfaction after TKA [2, 3]. Studies reporting the outcome of TKA also should report sex-specific analysis in view of potential differences in outcome [4]. Conflicting evidence exists as to the influence of sex on the outcome of TKA, with some authors reporting worse postoperative functional outcomes and lower satisfaction rates in females [5–7], whereas others report no difference [8, 9].

Female patients generally report worse preoperative pain and functional scores relative to males prior to their TKA. This may be related to females choosing to delay joint replacement and subsequent progression and worsening of their symptoms [10]. In addition to these worse preoperative functional scores, female patients are more likely to have lower back pain [11], depression [11], worse preoperative mental health [12] and more comorbidities [11], which are all associated with a worse functional outcome and lower rates of satisfaction after TKA [9, 12–14]. When adjustments are made for confounding factors between the sexes, no significant difference in pain relief was achieved after TKA [11, 15], but the functional outcome remains worse for females [7]. Whether this functional deficit is clinically significant remains unknown [16]. The influence of sex on patient satisfaction is also not clear, with studies adjusting for confounding variables demonstrating contrasting results [5, 9, 17].

The primary aim of this study was to assess whether sex had a clinically significant independent influence on the outcome of TKA according to the Western Ontario and McMaster Universities Osteoarthritis Index (WOMAC) score. The secondary aims were to assess whether sex independently influenced the outcome of TKA according to the Short Form (SF)-12 scores and patient satisfaction at 1 year. The hypothesis was that female patients would have a worse functional improvement and lower rate of satisfaction after TKA.

## Methods

**Fig. 1:**
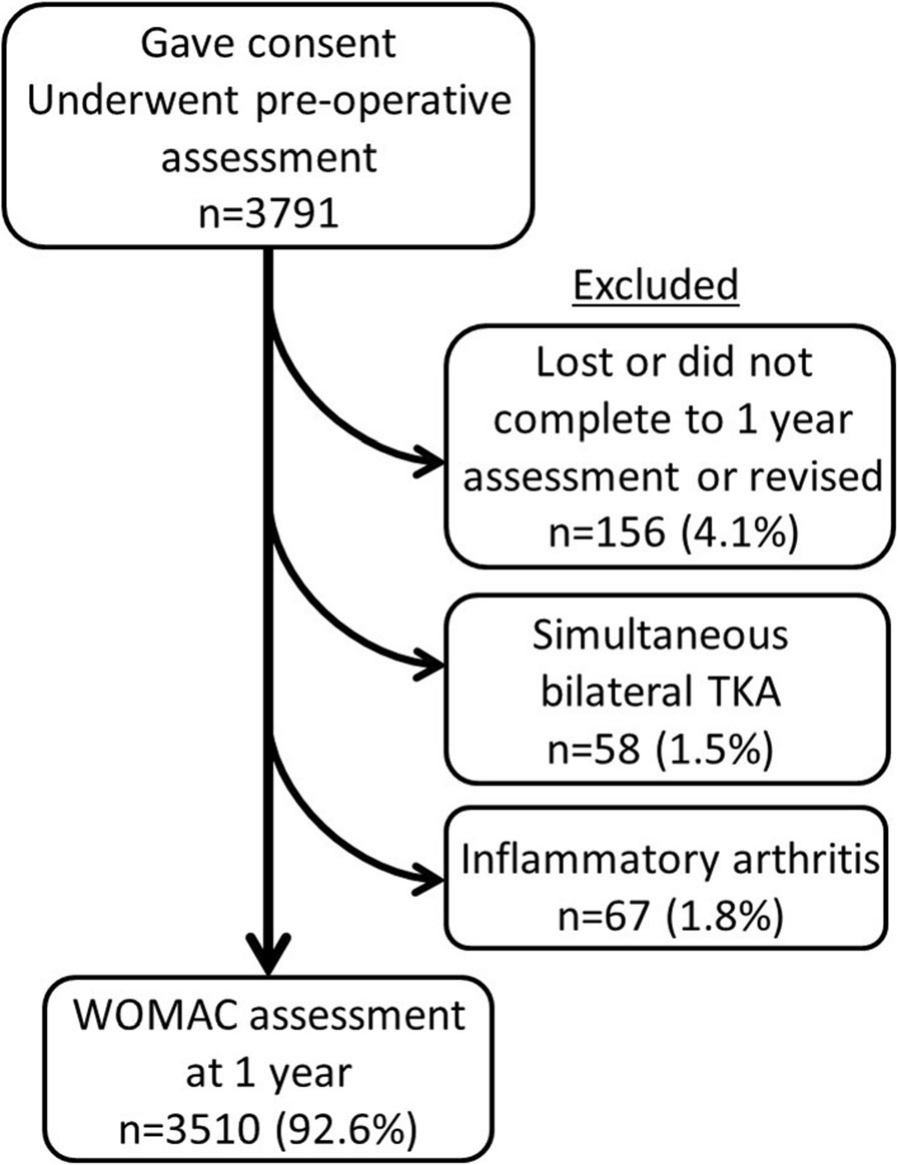
STROBE flow diagram for patient enrolment into the study

Patients for this study were identified retrospectively from a prospectively compiled arthroplasty database held at the study centre. During a 14-year period, 3791 patients undergoing primary TKA at the study centre were asked to complete a preoperative patient questionnaire. The inclusion criterion was completion of the preoperative questionnaire. Exclusion criteria were not completing the 1-year WOMAC score, undergoing simultaneous bilateral TKA or undergoing a TKA for inflammatory arthritis. The Strengthening the Reporting Observational studies in Epidemiology (STROBE) flow diagram of patient enrolment is illustrated in Fig. 1. There were 3510 TKA performed during the study period with complete pre- and postoperative data that met the inclusion criteria. There were 1584 male patients and 1926 female patients, with a combined mean age of 69.2 (standard deviation (SD) 9.7) years.

Patient demographics, body mass index (BMI) and comorbidities were recorded preoperatively. Comorbidities were recorded as a categorical yes or no for heart disease, hypertension, lung disease, diabetes, stomach ulcer, kidney disease, liver disease, anaemia, cancer, depression, neurological disease and back pain. The WOMAC [18] score and SF-12 score [19] were assessed preoperatively and 1-year postoperatively.

The WOMAC [18] used in this study was the Likert version 3.1, standardized with English for a British population, consisting of 24 self-administrated questions that were answered for each item on a 5-point Likert scale (none, mild, moderate, severe and extreme). It was reported as three separate subscales: pain, physical function, and stiffness. The WOMAC pain subscale had five questions scored 0 to 4 and was considered invalid if more than one item was missing; hence, it had a range from 0 (no pain) to 20 (maximal pain). In the event of a missing item, the remaining four items were averaged and then multiplied by 5 [20]. The WOMAC function subscale has 17 questions scored 0–4 and was considered invalid if more than three items were missing. It had a range of 0 (maximal function) to 68 (minimal function). In the event of missing items, the remaining items were averaged and then multiplied by 17. The WOMAC stiffness subscale had two items scored 0–4 and was considered invalid if either was missing; hence, it had a range from 0 (no stiffness) to 8 (maximal stiffness). The final scores were determined by adding the corresponding items for each dimension and then standardising to a range of values from 0 to 100. According to recent recommendations a reverse score was used from 0 (worst) to 100 (best) [21]. The minimal important change (MIC) in the WOMAC was defined as 21 for pain, 16 for function and 13 for stiffness, and the minimum clinically important difference (MCID) was defined as 11 for pain, 9 for function and 8 for stiffness [16].

The SF-12 is a generic assessment tool to measure a patient’s wellbeing, which is assessed using a physical component summary (PCS) and a mental component summary (MCS) score [19]. The SF-12 PCS and MCS scores range from 0 (worst level of functioning) to 100 (best level of functioning). The MCID was defined as 1.8 and MIC 2.7 for the SF-12 PCS score [22].

Patient satisfaction was assessed 1 year after surgery using four questions with a different focus:

1. “Overall, how satisfied are you with the results of your knee replacement surgery?”
2. “How satisfied are you with the results of your knee replacement surgery for improving your ability to do housework or yard work (such as cooking, cleaning, or gardening and raking leaves)?”
3. “How satisfied are you with the results of your knee replacement surgery for improving your ability to do recreational activities (such as taking walks, swimming, bicycling, playing golf, dancing, going out with friends)?”
4. “How satisfied are you with the results of your knee replacement surgery for relieving your pain?”

The response to the question was recorded using a four point Likert scale: very satisfied, somewhat satisfied, somewhat dissatisfied and very dissatisfied. This question with the four-point Likert assessment have been validated and demonstrated to be reliable to measure satisfaction after TKA [23]. Patients stating they were very satisfied and satisfied were categorised as satisfied, and those who defined their outcome as dissatisfied or very dissatisfied were categorised as dissatisfied [24].

### Statistical analysis

Statistical analysis was performed using Statistical Package for Social Sciences version 17.0 (SPSS Inc., Chicago, IL, USA). The data demonstrated a normal distribution, and parametric tests were used to assess continuous variables for significant differences between groups. Unpaired and paired Student’s t-tests were used to compare linear variables between groups. Dichotomous variables were assessed using a chi-square test. Linear regression analyses were used to identify independent preoperative predictors of change in the components of the WOMAC scores at 1 year. Linear (WOMAC and SF-12) and logistic (satisfaction) regression analyses were used to identify independent preoperative predictors of change in score and satisfaction at one year. A *p* value of < 0.05 was defined as statistically significant.

No additional patient contact was required, and as such, this project was performed as a service evaluation without the need for formal ethical approval. The project was registered with the institutions audit department (Newcastle Hospitals NHS Foundation Trust, Project Record Number 3290) and was conducted in accordance with the Declaration of Helsinki and the guidelines for good clinical practice.

## Results

Female patients had a significantly higher BMI (*p* < 0.001) and were less likely to suffer from heart disease (*p* < 0.001), diabetes (*p* = 0.04) and cancer (*p* = 0.03) but more likely to suffer from lung disease (*p* = 0.010), gastric ulceration (*p* < 0.001), anaemia (*p* = 0.001), back pain (*p* < 0.001) and depression (*p* < 0.001) when compared to male patients (Table 1). All preoperative functional measures were significantly (*p* ≤ 0.001) worse in female patients but were not greater than the MCID for the WOMAC or SF-12 scores (Table 1).

**Table 1:** Patient demographics and preoperative functional scores according to sex for the study cohort (*n* = 3510)

Demographic	Description	Sex		Difference / Odds Ratio (95% CI)	*P*-value*
		Male (*n* = 1584)	Female (*n* = 1926)		
**Age** (years: mean, SD)		69.4 (9.1)	69.2 (10.2)	Diff 0.2 (−0.5 to 0.8)	0.65
**BMI** (Kg/M^2^: mean, SD)		29.4 (4.5)	30.5 (7.7)	Diff 1.1 (−0.7 to 1.6)	< 0.001
**Comorbidity** (*n*, % of group)	Heart Disease (*n* = 538)	322 (20.3)	216 (11.2)	OR 0.5 (0.4 to 0.6)	< 0.001**
	Hypertension (*n* = 1726)	802 (50.6)	924 (48.0)	OR 0.9 (0.8 to 1.0)	0.12**
	Lung disease (*n* = 499)	200 (12.6)	299 (15.5)	OR 1.3 (1.1 to 1.5)	0.01**
	Neurological disease (*n* = 186)	91 (5.7)	95 (4.9)	OR 0.9 (0.6 to 1.1)	0.29**
	Diabetes mellitus (*n* = 398)	199 (12.6)	199 (10.3)	OR 0.8 (0.7 to 1.0	0.04**
	Gastric ulceration (*n* = 419)	137 (8.6)	282 (14.6)	OR 1.8 (1.5 to 2.2)	< 0.001**
	Kidney disease (*n* = 94)	39 (2.5)	55 (2.9)	OR 1.2 (0.8 to 1.8)	0.47**
	Liver disease (*n* = 48)	25 (1.6)	23 (1.2)	OR 0.7 (0.4 to 1.3)	0.33**
	Anaemia (*n* = 205)	69 (4.4)	136 (7.1)	OR 1.7 (1.2 to 2.2)	0.001**
	Cancer (*n* = 156)	84 (5.3)	72 (3.7)	OR 0.69 (0.5 to 0.9)	0.03**
	Back pain (*n* = 1572)	593 (37.4)	979 (50.8)	OR 1.7 (1.5 to 2.0)	< 0.001**
	Depression (*n* = 444)	128 (8.1)	316 (16.4)	OR 2.2 (1.8 to 2.8)	< 0.001**
**WOMAC** (mean, SD)	Pain	38.7 (17.9)	32.7 (17.5)	Diff 6.0 (4.9 to 7.2)	< 0.001
	Function	40.1 (18.0)	34.5 (16.7)	Diff 5.7 (4.5 to 6.8)	< 0.001
	Stiffness	41.6 (34.2)	34.2 (19.2)	Diff 7.4 (6.0 to 8.7)	< 0.001
	Total	40.0 (16.9)	34.0 (15.9)	Diff 5.9 (4.8 to 7.0)	< 0.001
**SF-12** (mean, SD)	Physical	28.6 (7.7)	27.7 (7.5)	Diff 0.9 (0.4 to 1.4)	0.001
	Mental	48.7 (13.2)	45.8 (13.6)	Diff 2.9 (2.0 to 3.8)	< 0.001

*unpaired Students t-test unless otherwise stated, **chi square test

Both male and female patients had a statistically (*p* < 0.001) and clinically (greater than the MIC) significant improvement in their WOMAC and SF-12 scores at 1 year (Table 2). Female patients had a statistically significantly lower 1-year WOMAC and SF-12 scores relative to males, but these were not clinically significant (Table 2). Conversely, female patients had a statistically significant (*p* < 0.001) greater overall improvement in the component and total WOMAC scores, but again, this was not clinically significant (Table 2). When adjustments were made for confounding differences, females had a statistically significant greater improvement in the their WOMAC function (*p* = 0.03) and total (*p* = 0.03) scores compared to males, but these improvements were not clinically significant (Table 3).

**Table 2:** Postoperative outcome measures and the difference relative to preoperative scores for the all patients according to sex

Functional measure		Sex		Difference (95% CI)	*P*-value*
		Male (*n* = 1584)	Female (*n* = 1926)		
**WOMAC**					
**Pain**	**One year** (SD)	79.8 (22.5)	77.8 (24.2)	2.0 (0.4 to 3.5)	0.01
	**Change** (95% CI)	41.1 (39.9 to 42.4)	45.2 (43.9 to 46.4)	4.0 (2.2 to 5.8)	< 0.001
	*p*-value**	< 0.001	< 0.001		
**Function**	**One year** (SD)	75.8 (20.6)	74.2 (21.5)	1.6 (0.2 to 3.0)	0.03
	**Change** (95% CI)	35.7 (34.6 to 36.7)	39.7 (38.7 to 40.6)	4.0 (2.5 to 5.5)	< 0.001
	*p*-value**	< 0.001	< 0.001		
**Stiffness**	**One year** (SD)	73.3 (21.8)	71.6 (23.4)	1.8 (0.2 to 3.3)	0.02
	**Change** (95% CI)	31.9 (30.9 to 33.2)	37.4 (36.2 to 38.5)	5.5 (3.7 to 7.2)	< 0.001
	*p*-value**	< 0.001	< 0.001		
**Total**	**One year** (SD)	76.8 (19.5)	75.3 (20.3)	1.5 (0.1 to 2.8)	0.03
	**Change** (95% CI)	36.9 (35.9 to 38.0)	41.2 (40.3 to 42.2)	4.3 (2.9 to 5.8)	< 0.001
	*p*-value**	< 0.001	< 0.001		
**SF-12**					
**PCS**	**One year** (SD)	39.4 (11.1)	37.5 (11.3)	1.9 (1.2 to 2.7)	< 0.001
	**Change** (95% CI)	10.8 (10.3 to 11.4)	9.8 (9.3 to 10.3)	1.1 (0.4 to 1.8)	0.003
	*p*-value**	< 0.001	< 0.001		
**MCS**	**One year** (SD)	50.5 (12.7)	48.2 (13.5)	2.3 (1.4 to 3.1)	< 0.001
	**Change** (95% CI)	1.8 (1.2 to 2.4)	2.4 (1.8 to 3.0)	0.6 (−0.2 to 1.5)	0.14
	*p*-value**	< 0.001	< 0.001		

* t-test

** paired t-test

**Table 3:** Multivariable linear regression analysis was used to identify the independent effect of sex on change in the components of the WOMAC and SF-12 scores 1 year after TKA. All variables (in Table 1) were all entered into the model at the one stage using “enter” methodology

Functional measure		B	95% Confidence intervals		*P*-value
**WOMAC**					
**Pain** (*R*^2^ = 0.29)					
**Sex**	Male	Reference			
	Female	0.9	−0.7	2.5	0.27
**Function** (*R*^2^ = 0.26)					
**Sex**	Male	Reference			
	Female	1.5	0.2	2.9	0.03
**Stiffness** (*R*^2^ = 0.36)					
**Sex**	Male	Reference			
	Female	1.2	−0.3	2.8	0.12
**Total** (*R*^2^ = 0.27)					
**Sex**	Male	Reference			
	Female	1.5	0.2	2.8	0.03
**SF-12**					
**PCS** (*R*^2^ = 0.19)					
**Sex**	Male	Reference			
	Female	−0.4	−1.1	0.3	0.21
**MCS** (*R*^2^ = 0.30)					
**Sex**	Male	Reference			
	Female	−0.2	−0.9	0.6	0.70

A higher rate of overall satisfaction was observed in male patients, with a trend towards significance (*p* = 0.054) for female patients to be less likely to be satisfied (Table 4). Female patients were significantly less likely to be satisfied with pain relief (*p* = 0.005), return to work (*p* = 0.003) and return to recreational activities (*p* = 0.02) compared to male patients (Table 4). However, when adjustments were made for confounding differences, only satisfaction with pain relief remained significantly (*p* = 0.03) less likely for females relative to males (Table 5).

**Table 4:** Overall patient satisfaction and satisfaction with pain relief, return to work and recreational activities 1 year after TKA according to sex

Satisfaction	Sex (n, % of group)		OR	95% CI	*P*-value
	Male	Female			
**Overall**					
Satisfied	1429 (90.7)	1701 (88.7)	0.81	0.65 to 1.00	0.054
Dissatisfied	146 (9.3)	216 (11.3)			
**Pain**					
Satisfied	1448 (91.8)	1704 (88.9)	0.72	0.57 to 0.91	0.005
Dissatisfied	130 (8.2)	212 (11.1)			
**Work**					
Satisfied	1386 (87.9)	1622 (84.4)	0.74	0.61 to 0.90	0.003
Dissatisfied	190 (12.1)	300 (15.6)			
**Recreation**					
Satisfied	1300 (82.1)	1518 (78.8)	0.81	0.69 to 0.96	0.02
Dissatisfied	284 (17.9)	408 (21.2)

**Table 5:** Multivariable logistic regression analysis was used to identify the independent effect of sex on overall patient satisfaction and satisfaction with pain relief, return to work and recreation activities one year after TKA. All variables (in Table 1) were all entered into the model at the one stage using “enter” methodology

Functional measure		OR	95% Confidence intervals		*P*-value
**Overall** (*R*^2^ = 0.07)					
**Sex**	Male	Reference			
	Female	0.88	0.68	1.13	0.30
**Pain** (*R*^2^ = 0.07)					
**Sex**	Male	Reference			
	Female	0.75	0.58	0.98	0.03
**Work** (*R*^2^ = 0.10)					
**Sex**	Male	Reference			
	Female	0.84	0.67	1.05	0.13
**Recreation** (*R*^2^ = 0.12)					
**Sex**	Male	Reference			
	Female	0.94	0.77	1.14	0.94
	Female	−0.2	−0.9	0.6	0.70

## Discussion

This study has demonstrated that female patients have significantly different preoperative comorbidities, more severe knee pain and worse function, and worse generic physical and mental health scores compared to male patients. Although, female patients had a greater overall improvement in the joint-specific WOMAC function and pain scores and had lower 1-year WOMAC and SF-12 scores relative to males, these differences were not clinically meaningful. A lower rate of satisfaction with pain relief, return to work and return to recreational activities was observed for female compared to male patients, but only satisfaction with pain relief remained significantly less likely for females when adjustments were made for confounding.

The retrospective design of this study is a limitation, and additional information such as patient expectations and radiographic severity and pattern of the arthritis affecting the knee were not obtained. Whether patient expectations were achieved after TKA and how sex influences the achievement of these may have given some insight to the reasons for the lower rate of satisfaction with pain relief. Also, the radiographic severity of the arthritis in the knee was not assessed, and this would have allowed a correlation to be made with the subjectively worse pain and functional WOMAC scores observed in female patients, thereby allowing determination of whether these worse scores correlate with more severe degenerative changes. The assessment of associated comorbidity was also a limitation, being simply recorded as present or not, with no grading of severity, which may have influenced the pre- and postoperative functional outcome and satisfaction rate.

This study has affirmed that female patients have statistically significantly worse preoperative knee specific function and pain prior to their TKA compared to male patients, which has been observed previously [6, 11, 15, 25, 26]. The novel aspect of the current study was the application of the MCID as a clinically important threshold, and females patients were not clinically different from male patients preoperatively. A similar pattern was demonstrated in the postoperative WOMAC scores for the study cohort, being worse for female relative to male patients, but these differences were not clinically significant. Other authors have demonstrated similar findings in the postoperative score when presented as an absolute difference [6, 25, 26]. However, when they adjusted for confounding variables, Mehta et al. [11] and Perruccio et al. [15] found no difference in the postoperative scores, whereas Escobar et al. [7] found that female patients scored 5 points worse in the function and stiffness components of the WOMAC score 6 months postoperatively. The current study supports this statistically significant difference in the function component of the WOMAC score between female and male patients postoperatively when adjusting for confounding; however, this difference was not greater than the MCID of 9 points and does not represent a clinically meaningful difference. Other factors more prevalent in female patients may result in a clinically significant influence on outcome after TKA, such as depression and back pain [9, 12, 13].

The rate of patient satisfaction after TKA was lower in female patients relative to male patients in the current study; however, after adjustments had been made for confounding variables, only satisfaction with pain relief remained significantly less likely in females. Why this should be is not clear in view of equal improvement and no clinically significant difference in the postoperative WOMAC pain scores observed in the study cohort between male and female patients. Female patients in this study were shown to have a greater BMI and prevalence of depression and back pain relative male patients, all of which have been associated with a lower rate of patient satisfaction after TKA [9, 13, 27]. The reason for these pathologies being more prevalent in females is not clear but may relate to pain catastrophising, which is more prevalent in females [28, 29]. A meta-analysis found pain catastrophising to be associated with persistent knee pain after TKA [30]. Contralateral knee pain is also associated with a lower of satisfaction after TKA and was not accounted for in the current study and may have been more prevalent in females who are more likely to have bilateral symptoms [31]. However, similar to the current study, other authors have demonstrated, once the confounding differences are adjusted for between male and female patients, no difference existed in the rate of overall satisfaction [9, 17, 32, 33]. Data from the National Joint Registry suggests that female patients are more likely to be dissatisfied after TKA [5]; however, those data were adjusted for some confounding factors but not for some factors more common in female patients such as the depression, back pain and worse preoperative mental health.

No difference existed in age between the female and male patients in the current study. This is not in keeping with prior comparative studies, with some studies finding female patients to be 1 to 3 years older at the time of their TKA and concluding a delay to surgery [11, 15, 34]. The reason(s) for this age difference/delay is not clear, but the findings of the current may support these previous studies in part, with the worse preoperative function and pain scores being observed in females suggesting worse disease at the time of TKA and may be related to a delay in presentation. Other authors have suggested the delay may be due to selection bias of orthopaedic surgeons when listening for TKA [11, 15]. Studies by Borkhoff et al. [35, 36] demonstrated that family physicians and orthopaedic surgeons were less likely to recommend a female patient for a TKA compared to a male patient. This may have resulted in female patients being delayed in referral by the physician and also by orthopaedic surgeon for listening for TKA, which may explain in part the worse pain and functional status relative to males preoperatively.

## Conclusion

Sex does not clinically influence the knee-specific outcome (WOMAC) or overall generic (SF-12) health 1 year after TKA. However, satisfaction with pain relief after TKA was significantly less likely in female patients.

## Data Availability

Data is available on request to the corresponding authors should it be required, but this would have to be authorised by the study centre audit team.
